# Feasibility, Usability, and Effectiveness of a Machine Learning–Based Physical Activity Chatbot: Quasi-Experimental Study

**DOI:** 10.2196/28577

**Published:** 2021-11-26

**Authors:** Quyen G To, Chelsea Green, Corneel Vandelanotte

**Affiliations:** 1 Physical Activity Research Group Appleton Institute Central Queensland University Rockhampton Australia

**Keywords:** conversational agent, virtual coach, intervention, exercise, acceptability, mobile phone

## Abstract

**Background:**

Behavioral eHealth and mobile health interventions have been moderately successful in increasing physical activity, although opportunities for further improvement remain to be discussed. Chatbots equipped with natural language processing can interact and engage with users and help continuously monitor physical activity by using data from wearable sensors and smartphones. However, a limited number of studies have evaluated the effectiveness of chatbot interventions on physical activity.

**Objective:**

This study aims to investigate the feasibility, usability, and effectiveness of a machine learning–based physical activity chatbot.

**Methods:**

A quasi-experimental design without a control group was conducted with outcomes evaluated at baseline and 6 weeks. Participants wore a Fitbit Flex 1 (Fitbit LLC) and connected to the chatbot via the Messenger app. The chatbot provided daily updates on the physical activity level for self-monitoring, sent out daily motivational messages in relation to goal achievement, and automatically adjusted the daily goals based on physical activity levels in the last 7 days. When requested by the participants, the chatbot also provided sources of information on the benefits of physical activity, sent general motivational messages, and checked participants’ activity history (ie, the step counts/min that were achieved on any day). Information about usability and acceptability was self-reported. The main outcomes were daily step counts recorded by the Fitbit and self-reported physical activity.

**Results:**

Among 116 participants, 95 (81.9%) were female, 85 (73.3%) were in a relationship, 101 (87.1%) were White, and 82 (70.7%) were full-time workers. Their average age was 49.1 (SD 9.3) years with an average BMI of 32.5 (SD 8.0) kg/m2. Most experienced technical issues were due to an unexpected change in Facebook policy (93/113, 82.3%). Most of the participants scored the usability of the chatbot (101/113, 89.4%) and the Fitbit (99/113, 87.6%) as at least “OK.” About one-third (40/113, 35.4%) would continue to use the chatbot in the future, and 53.1% (60/113) agreed that the chatbot helped them become more active. On average, 6.7 (SD 7.0) messages/week were sent to the chatbot and 5.1 (SD 7.4) min/day were spent using the chatbot. At follow-up, participants recorded more steps (increase of 627, 95% CI 219-1035 steps/day) and total physical activity (increase of 154.2 min/week; 3.58 times higher at follow-up; 95% CI 2.28-5.63). Participants were also more likely to meet the physical activity guidelines (odds ratio 6.37, 95% CI 3.31-12.27) at follow-up.

**Conclusions:**

The machine learning–based physical activity chatbot was able to significantly increase participants’ physical activity and was moderately accepted by the participants. However, the Facebook policy change undermined the chatbot functionality and indicated the need to use independent platforms for chatbot deployment to ensure successful delivery of this type of intervention.

## Introduction

### Background

It has been established that physical activity reduces the risk of mortality and many health conditions such as cardiovascular diseases, type 2 diabetes, and cancer [1]. However, less than half of Australian adults meet the physical activity guidelines of at least 150 minutes of vigorous-moderate-intensity physical activity per week [2]. It was estimated that physical inactivity accounted for 53.8 billion in health care costs and an additional 13.7 billion in productivity losses worldwide in 2013 [3]. Therefore, interventions to increase physical activity are needed [4]. To date, many of these interventions have been delivered face-to-face and are expensive [5]. Therefore, there is a need for low-cost interventions targeting large populations.

With the advancement of mobile technology, people can access the internet almost everywhere and at any time. It is estimated in 2019 that 4.48 billion people are active internet users, 4.07 billion are unique mobile internet users, and 3.66 billion are active mobile social media users [6]. This indicates that mobile health (mHealth) has the potential to offer a great platform for behavior change interventions that can reach a large number of people at a low cost. In the last decade, many eHealth and mHealth interventions targeting physical activity have been examined [7-10], many of which use email, SMS, and websites as delivery tools. Overall, these interventions have been able to produce moderate effect sizes in increasing physical activity [7-10]. As such, there is still room to further increase the effectiveness of behavioral eHealth and mHealth interventions. One often cited problem in this area is the low levels of engagement and interaction with eHealth and mHealth interventions [11]. As there is evidence that the more participants use the interventions, the more effective the interventions tend to be [5,12], an important aim is to design eHealth and mHealth interventions that will lead to higher levels of engagement.

The use of chatbots is a potential innovative avenue for achieving higher levels of engagement. A chatbot or conversational agent is a computer program that can interact with users [13]. Equipped with natural language processing capability, a modern chatbot can effectively engage in conversations with users [14]. Chatbots can help save human resources while providing instant responses to requests. In particular, chatbots can also help users monitor participants’ progress by continuously evaluating physical activity data from wearable sensors and smartphones. Applying machine learning algorithms can also enable chatbots to provide personalized activity recommendations to a specific user. Chatbots can be embedded into different platforms, such as websites, apps, messaging programs or other social media to reach large numbers of people easily and conveniently. As such, chatbots have been adopted across many industries, such as finance, e-commerce, and health care [14-16].

Recent reviews indicate that health behavior change interventions using chatbots have mostly focused on mental health [17,18]. Among the few studies that used chatbots to promote physical activity and healthy diet [19], only 2 evaluated increases in physical activity [20,21]. However, the study conducted in Switzerland was designed to test differences in daily step goals among 3 groups (cash incentives vs charity incentives vs no incentives) rather than the effectiveness of the chatbot [21]. Only one study in Australia evaluated the effectiveness of a chatbot in improving diet and physical activity [20]. Although this study focused more on diet than physical activity, it did show a large increase in physical activity (approximately 110 moderate-to-vigorous physical activity min/week). However, the chatbot evaluated in this study did not provide automatic daily updates that remind the participants about their physical activity goals and did not automatically adjust participants’ goals based on their current physical activity level.

### Objectives

Given the lack of studies on the effectiveness of physical activity chatbots, the aim of this study is to investigate the feasibility, usability, and effectiveness of an interactive machine learning–based physical activity chatbot that uses natural language processing and adaptive goal setting.

## Methods

### Study Design and Participants

A quasi-experimental design without a control group was conducted with outcomes evaluated at 2 time points—baseline and 6 weeks after participants started to use the chatbot. Prospective participants were recruited from a list of people who had previously used the 10,000 Steps program [22]. To be eligible, potential participants had to be inactive (<20 min/day of moderate-to-vigorous physical activity), live in Australia, have internet access and a smartphone, aged at least 18 years, self-reported motivation to improve physical activity (targeting those in need of support to become more active), not already participating in another physical activity program, not already owning and used a physical activity tracking device (eg, pedometer, Fitbit [Fitbit LLC], and Garmin) within the last 12 months, and able to safely increase their activity levels. Those who were interested in the study and clicked on the link attached to the invitation emails were directed to a web-based survey. Prospective participants were provided with a participant information sheet and contact details of the research team and then asked to answer a series of screening questions to assess eligibility. If eligible, they completed a web-based consent form and baseline survey questions. After 6 weeks, the participants were asked to complete a follow-up web-based survey to assess changes over time.

Owing to an unexpected Facebook policy change (we used the Messenger app to host the chatbot, which is owned by Facebook) that blocked the chatbot from sending out new messages to participants who did not respond to the previous message within 24 hours, we were forced to stop the study at that point. As the recruitment was rolling, 48 participants had already completed the study when the Facebook policy change was implemented. For those who were still engaged in the study at that time, a follow-up survey was sent to them immediately at the time of implementation of this policy, resulting in a shorter intervention period.

**Figure 1:**
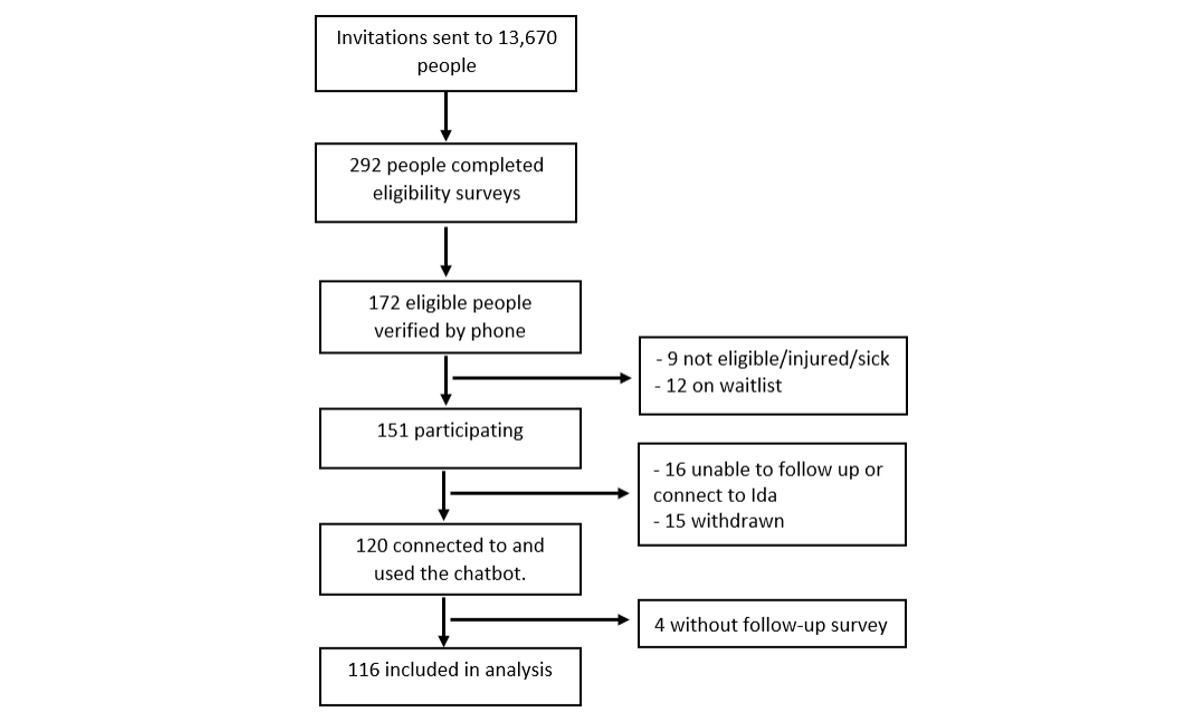
Participant flowchart.

Invitation emails were sent to 13,670 email addresses registered in the 10,000 Steps program database between September and November 2020 (Figure 1). A total of 2.14% (292/13, 670) of people completed the eligibility survey during the recruitment period, with 58.9% (172/292) people deemed eligible. Eligible people were contacted by phone for verification. This resulted in 9 people being excluded from the study because they did not meet the eligibility criteria upon verification, were no longer interested, or had an illness or injury that prevented them from taking part. When recruitment closed, 12 people were placed on a waitlist. Another 16 people were excluded because they were unable to be recontacted or to connect to the chatbot, and 15 withdrew because of an illness or personal issues. As a result, 120 participants were enrolled at baseline. However, only 116 completed the follow-up surveys.

This study was approved by the Human Research Ethics Committee of the Central Queensland University (application #0000022181). This study was retrospectively registered on the Australian New Zealand Clinical Trials Registry (ACTRN12621000345886).

### Procedures

Participants who agreed to participate, provided written consent, and completed the baseline survey were mailed a package including a Fitbit Flex 1 activity tracker (with instructions on how to use it), a participant information sheet, and instructions on how to download the Fitbit app on their smartphone and how to create a Fitbit account. Follow-up phone calls were conducted to ensure that participants received the package and were able to install the Fitbit app and use the Fitbit device.

Participants wore their Fitbit for 7 days to collect their baseline physical activity data before connecting to the chatbot. To connect to the chatbot, participants were instructed to download and open the Messenger app on their smartphones and complete the secure verification process (only study participants were able to connect with the chatbot). Once verified, the participants started to receive daily messages and were able to interact with the chatbot. Participants were asked to engage with the chatbot (intervention) for a period of 6 weeks.

Follow-up surveys were sent to the participants via email. Four reminders (a combination of text messages, email, and phone calls), each of which was 3 days apart, were sent to ask participants to complete the follow-up survey. A research assistant was available during the intervention period to assist participants with any technical issues that the participants may encounter.

### Intervention

The chatbot, named *Ida,* was created and technically managed by an Australian company called SmartAI. However, the natural language processing capability was powered by Dialogflow (Google Inc), an advanced Google machine learning platform for creating conversational artificial intelligence applications. The Facebook Messenger app was selected for the deployment of the chatbot because of its popularity. The Fitbit Flex 1 device was used to measure the participants’ daily physical activity. Fitbit activity data were synced from the Fitbit platform to the chatbot platform and used by the chatbot to monitor the participants’ progress over time.

The intervention was designed using the COM-B model. The COM-B model forms the core of the Behavior Change Wheel, a behavioral system focusing on 3 components: capability, opportunity, and motivation [23]. As explained below, the messages delivered by the chatbot aimed to increase participants’ motivation (ie, motivational messages), capability (ie, through ongoing adaptive feedback on goal achievement), and opportunity (ie, educational content and activity reminders throughout the day helped participants become more aware of physical activity opportunities). The chatbot supported participants through 2 groups of actions: proactive and reactive.

Proactive actions include the following: (1) Providing an update on participants’ physical activity level achieved the previous day and informing them of the goal they needed to achieve on the current day. This message was sent early in the morning at the time selected by each participant. (2) Sending out 1 or 2 additional messages later in the day to encourage participants trying to achieve their daily goal or indicate they were doing great and had already achieved the goal when the message was sent. The number of messages and times was selected by each participant. (3) Automatically adjusting the daily activity goals based on the average physical activity level achieved during the 7 previous days. The type of goal (step counts or minutes) and the amount per day (eg, 8000 steps/day or 35 min/day) that the participant wanted to achieve by the end of the study was also chosen by each participant. The goal was automatically adjusted to increase by 500 steps/day or 5 minutes of moderate-vigorous physical activity/day if the participant, on average, met their current goal over the last 7 days [24,25]. If not, the same goal was used. We used a combination of moderate and vigorous physical activity assessed by Fitbit to calculate the physical activity min/week. The information needed to personalize the chatbot was collected during the verification phone calls and added to the participants’ profile page on the chatbot platform, which was only accessible to the research team.

**Figure 2:**
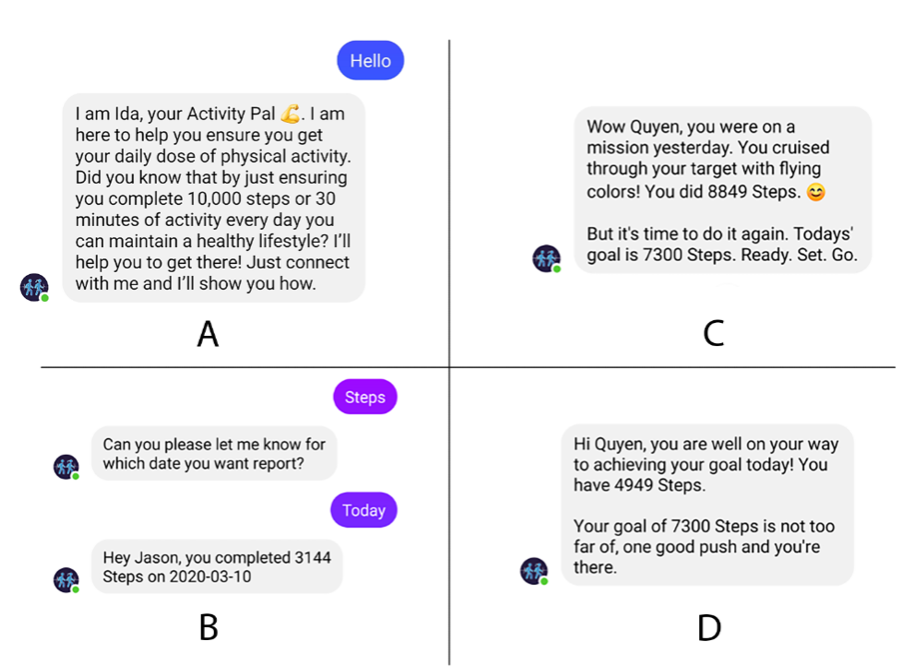
Message examples: (A) introduction, (B) request on step counts, (C) message upon reaching the goal, (D) message encouraging the participant to try reaching the goal.

Reactive actions, which occurred when the participants sent a request for information to the chatbot, include (1) providing sources of information on the benefits of physical activity, (2) sending general motivational messages to encourage participants to become more active, and (3) checking participants’ activity history (ie, the step counts or minutes that were achieved on any day) as requested. Examples of these messages are shown in Figure 2.

### Measures

Demographic characteristics were self-reported at baseline. Age, height, weight, years of schooling, and average daily work time (hours) were used as continuous variables; categorical variables included gender (male or female), marital status (not in a relationship or in a relationship), ethnicity (White or other), living area (major city, regional, or remote area), work status (full-time or other), and annual household income (≥Aus $130,000 [US $94,900], Aus $78,000 to <Aus $130,000 [US $56,940- $94900], or <Aus $78,000 [US $56,940]). Weight was also self-reported at follow-up. BMI was calculated as weight (kg)/height (m^2^) and was analyzed as a secondary outcome.

Physical activity was objectively measured using the Fitbit Flex 1. Although this device records both step counts and physical activity minutes, only step counts were used in the analysis. This is because the Fitbit only recorded the minutes if a user was active for at least 10 minutes, whereas all steps were counted regardless of whether they occurred during bouts of activity (10 minutes) or not.

Self-reported physical activity was assessed at baseline (before receiving a Fitbit) and follow-up using the Active Australia Survey [26]. These questions asked about minutes participants spent on walking, moderate and vigorous physical activity per day, and the number of days they spent engaging in these activities in the last week. The total amount of time spent engaging in walking and moderate and vigorous physical activity in a week was calculated by adding the above times (with vigorous physical activity time doubled as per scoring instructions) [26]. Participants who spent at least 150 minutes of moderate-to-vigorous physical activity per week were categorized as meeting the Australian physical activity guidelines.

Usability and acceptability were assessed at follow-up using the System Usability Scale (SUS) [27] and other self-reported questions. The SUS includes 10 questions with 5 response options from *strongly agree* to *strongly disagree*. As recommended, the original scores for each question were converted to new scores, summed, and multiplied by 2.5, to generate an SUS score between 0 and 100 [27]. We used the cutoffs suggested by Bangor et al [28] to classify the SUS scores into 4 groups: excellent (85.58-100), good (72.75-85.57), OK (52.01-72.74), and poor (0-52.00). Other self-reported questions asked about the usefulness of the chatbot, willingness to use the chatbot in the future and recommend it to others, whether a participant experienced any technical issues, Fitbit wear time, and frequency of using the Fitbit app.

### Power and Data Analysis

Posthoc power calculation was conducted for Fitbit step counts using the following parameters: difference in means, SDs, and correlation between step counts at 2 time points. The posthoc power for this study was 81.3%.

Fitbit data were cleaned and processed using the Python v3.7 (Python Software Foundation). As step counts of <1000 indicate that the Fitbit was not worn all day [29,30], these counts were removed. A 7-day moving average for the daily mean steps was generated and used to show changes in steps over the study period. Average step counts were also calculated for weeks with at least 4 days of valid data; however, only data in the first week as baseline data (before the date that participants connected to the chatbot) and the last week of participation as follow-up data were used in the outcome analysis. As participation duration was different among the participants, doing this allowed the analysis to be performed for all participants together.

SAS v9.4 (SAS Institute) was used for the analysis. Baseline characteristics were compared among those participating <4 weeks, 4 to <6 weeks, and ≥6 weeks using Fisher exact tests for categorical variables and Welch analysis of variance for continuous variables, except for daily work time and total physical activity minutes, which were tested using Kruskal–Wallis tests. As a robustness check, the analysis was performed separately for 2 samples, a full sample and a subsample (excluding those using the chatbot <4 weeks). This ensures that the results reflect the effectiveness of the intervention for those with sufficient exposure to the chatbot.

Generalized linear mixed models were used to identify changes in the outcomes. Normal distribution and identity link were used for BMI and Fitbit step counts. As total physical activity minutes were highly skewed, PROC TRANSREG was used to conduct the Box-Cox transformation analysis, and as a result, a fourth root transformation was applied. Generalized linear mixed models with normal distribution and log link were used for the transformed total physical activity minutes. Estimates were converted back into ratios for interpretative purposes. Empirical estimators were used to obtain the robust SEs. Binary distribution and logit link were used to determine the outcome of meeting physical activity guidelines. For each outcome, 2 models were run to generate crude estimates and estimates adjusted for sample characteristics including age, gender, marital status, years of schooling, ethnicity, household income, living area, work status, and daily work time. Differences in BMI, step counts, and total physical activity minutes between the follow-up and baseline were reported with a 95% CI. Odds ratios (ORs) and 95% CIs were reported for meeting the physical activity guidelines. All *P* values were 2-sided and considered significant if <.05.

## Results

### Baseline Characteristics

Table 1 presents the baseline characteristics of the sample. Most of the participants were female (95/116, 81.9%), in a relationship (85/116, 73.3%), White (101/116, 87.1%), and full-time workers (82/116, 70.7%). The participants had an average age of 49.1 (SD 9.3) years, with an average BMI of 32.5 (SD 8.0) kg/m^2^, and 81.9% (95/116) of the participants were either overweight or obese. The average step count was <6000 (SD 2391) steps/day. Only 13.8% (16/116) of the participants met the physical activity guideline. There were no significant differences in these characteristics among those with different participation durations (*P*>.05). Among the 116 participants, 17 participated in <4 weeks, 51 between 4 and <6 weeks, and 48 at least 6 weeks.

**Table 1:** Characteristics at baseline by participation duration (N=116).

			All (N=116)		<4 weeks (n=17)		4-<6 weeks (n=51)		At least 6 weeks (n=48)		*P* value	
**Gender, n (%)**												.49
	Male	21 (18.1)		2 (11.8)		12 (23.5)		7 (14.6)				
	Female	95 (81.9)		15 (88.2)		39 (76.5)		41 (85.4)				
**Marital status, n (%)**												.17
	Not in a relationship	31 (26.7)		7 (41.2)		15 (29.4)		9 (18.8)				
	In a relationship	85 (73.3)		10 (58.8)		36 (70.6)		39 (81.3)				
**Ethnicity, n (%)**												.27
	White	101 (87.1)		13 (76.5)		44 (86.3)		44 (91.7)				
	Others	15 (12.9)		4 (23.5)		7 (13.7)		4 (8.3)				
**Living areas, n (%)**												.72
	Major city	56 (48.3)		9 (52.9)		26 (51)		21 (43.8)				
	Regional or remote areas	60 (51.7)		8 (47.1)		25 (49)		27 (56.3)				
**Work status, n (%)**												.53
	Full-time	82 (70.7)		14 (82.4)		34 (66.7)		34 (70.8)				
	Others	34 (29.3)		3 (17.7)		17 (33.3)		14 (29.2)				
**Annual household income in Aus $ (US $), n (%)**												.85
	≥130,000 (≥94,900)	35 (30.2)		6 (35.3)		14 (27.5)		15 (31.3)				
	78,000 to <130,000 (56,940-94,900)	39 (33.6)		5 (29.4)		20 (39.2)		14 (29.2)				
	<78,000 (<56,940)	42 (36.2)		6 (35.3)		17 (33.3)		19 (39.6)				
Average age (years), mean (SD)			49.1 (9.3)		48.9 (11.0)		50.3 (9.0)		48.1 (9.0)		.48	
Average height (cm), mean (SD)			167.4 (8.9)		163.7 (11.6)		169.4 (9.2)		166.7 (7.0)		.11	
Average weight (kg), mean (SD)			91.3 (24.7)		94.3 (18.1)		91.3 (27.8)		90.3 (23.5)		.77	
Average BMI (kg/m^2^), mean (SD)			32.5 (8.0)		35.3 (6.7)		31.7 (8.6)		32.4 (7.7)		.19	
Average years of schooling, mean (SD)			15.8 (3.5)		15.5 (3.8)		15.6 (3.4)		16.0 (3.6)		.78	
Average daily work time (h/day), mean (SD)			8.0 (2.0)^a^		8.0 (1.9)		7.8 (1.8)^b^		8.0 (2.2)^c^		.97^d^	
Average step counts/day, mean (SD)			5933 (2391)^e^		5761 (2076)^f^		6466 (2800)^g^		5428 (1895)^h^		.12	
Average total physical activity (min/week), mean (SD)			86.5 (137.5)		72.4 (58.0)		91.4 (143.8)		86.3 (151.8)		.80^i^	
**Met physical activity recommendation, n (%)**												.12
	No	100 (86.2)		16 (94.1)		40 (78.4)		44 (91.7)				
	Yes	16 (13.8)		1 (5.9)		11 (21.6)		4 (8.3)				

^a^n=106.

^b^n=45.

^c^n=44.

^d^Fisher Exact tests or Welch analysis of variance was used unless indicated otherwise.

^e^n=108.

^f^n=14.

^g^n=48.

^h^n=46.

^i^Kruskal–Wallis test was used.

### Usability and Acceptability

Table 2 shows data on process evaluation on the implementation of the intervention. The average usability score for the chatbot was 61.6 (9.7) with majority of the participants scoring the chatbot as *OK* (89/113, 78.8%) or *good* (12/113, 10.6%). Although less than half would recommend the chatbot to others (49/113, 43.4%) and about one-third (40/113, 35.4%) would continue to use the chatbot in the future, more than half (60/113, 53.1%) agreed that the chatbot helped them become more active. About one-third thought the chatbot was quite or very useful in helping them increase confidence for engaging in regular physical activity, and in helping them stay motivated to participate in physical activity. About one-quarter thought the chatbot was useful in helping them overcome barriers, increase support they receive, and plan for physical activity during the study period. Most of the participants (106/113, 93.8%) read the messages that the chatbot sent out, and about half of the participants sent messages to the chatbot at least once a day. On average, the participants sent 6.7 messages to the chatbot per week and spent 5.1 minutes with the chatbot per day. About one-quarter liked very much the messages that the chatbot sent out. However, only 43.4% (49/113) thought that the chatbot understood their messages most of the time. Most participants experienced technical issues (93/113, 82.3%) and stopped receiving the chatbot messages at any time during the study (95/113, 84.1%).

**Table 2:** Usability and acceptability of the chatbot and Fitbit.

		Value
**System Usability Scale score for the chatbot (n=113), n (%)**		
	Good	12 (10.6)
	OK	89 (78.8)
	Poor	12 (10.6)
**Would recommend chatbot to others (n=113), n (%)**		
	Strongly agree or agree	49 (43.4)
	Neutral	36 (31.9)
	Strongly disagree or disagree	28 (24.7)
**Would continue to use the chatbot in future (n=113), n (%)**		
	Strongly agree or agree	40 (35.4)
	Neutral	32 (28.3)
	Strongly disagree or disagree	41 (36.3)
**The chatbot helped me to be more active (n=113), n (%)**		
	Strongly agree or agree	60 (53.1)
	Neutral	27 (23.9)
	Strongly disagree or disagree	26 (23)
**Usefulness—the chatbot helps me to increase confidence for physical activity participation (n=113), n (%)**		
	Not at all useful or a little useful	52 (46)
	Somewhat useful	27 (23.9)
	Quite useful or very useful	34 (30.1)
**Usefulness—the chatbot helps me to overcome barriers to physical activity participation (n=113), n (%)**		
	Not at all useful or a little useful	60 (53.1)
	Somewhat useful	24 (21.2)
	Quite useful or very useful	29 (25.7)
**Usefulness—the chatbot increased support for being active (n=113), n (%)**		
	Not at all useful or a little useful	59 (52.2)
	Somewhat useful	26 (23)
	Quite useful or very useful	28 (24.8)
**Usefulness—the chatbot helped me plan to be active (n=113), n (%)**		
	Not at all useful or a little useful	63 (55.7)
	Somewhat useful	20 (17.7)
	Quite useful or very useful	30 (26.6)
**Usefulness—the chatbot helped me to stay motivated (n=113), n (%)**		
	Not at all useful or a little useful	47 (41.6)
	Somewhat useful	26 (23)
	Quite useful or very useful	40 (35.4)
**Read the chatbot messages (n=113), n (%)**		
	Always	71 (62.8)
	Most of the time	35 (31)
	Sometimes or rarely	7 (6.2)
**Frequency of sending messages to chatbot (n=77), n (%)**		
	Several times a day	30 (26.6)
	Once a day	28 (24.8)
	Less than once a day	19 (48.6)
Average messages/week sent to the chatbot (n=113), mean (SD)		6.7 (7.0)
Average time/day spent with the chatbot (minutes; n=113), mean (SD)		5.1 (7.4)
**Liked the chatbot messages (n=113), n (%)**		
	Very much	26 (23)
	Average	42 (37.2)
	A little or not at all	45 (39.8)
**Understood the chatbot messages (n=113), n (%)**		
	Always or most of the time	49 (43.3)
	Sometimes	34 (30.1)
	Rarely or never	30 (26.5)
**Technical issues during the study (n=113), n (%)**		
	Yes	93 (82.3)
	No	20 (17.7)
**Chatbot stopped sending motivational messages or updates at any time (n=113), n (%)**		
	Yes	95 (84.1)
	No	18 (15.9)
**System Usability Scale for Fitbit Flex 1 (n=112), n (%)**		
	Good	22 (19.6)
	OK	77 (68.8)
	Poor	13 (11.6)
Average weeks of wearing the Fitbit (n=112), mean (SD)		5.4 (1.1)
Average day/week of wearing the Fitbit (n=112), mean (SD)		6.7 (0.9)
Average h/day of wearing the Fitbit (n=112), mean (SD)		19.5 (5.5)
**Frequency of using the Fitbit app (n=112), n (%)**		
	<1/day	19 (17)
	Once a day	27 (24.1)
	At least twice a day	66 (58.9)

The average usability for Fitbit was 64.0 (SD 11.1) with majority scoring the Fitbit usability as *OK* (77/113, 68.1%) or *Good* (22/113, 19.5%). More than half of the participants used the Fitbit app at least twice a day. On average, the participants wore the Fitbit for 5.4 weeks, 6.7 days per week, and 19.5 h/day.

### Effectiveness of the Intervention

**Figure 3:**
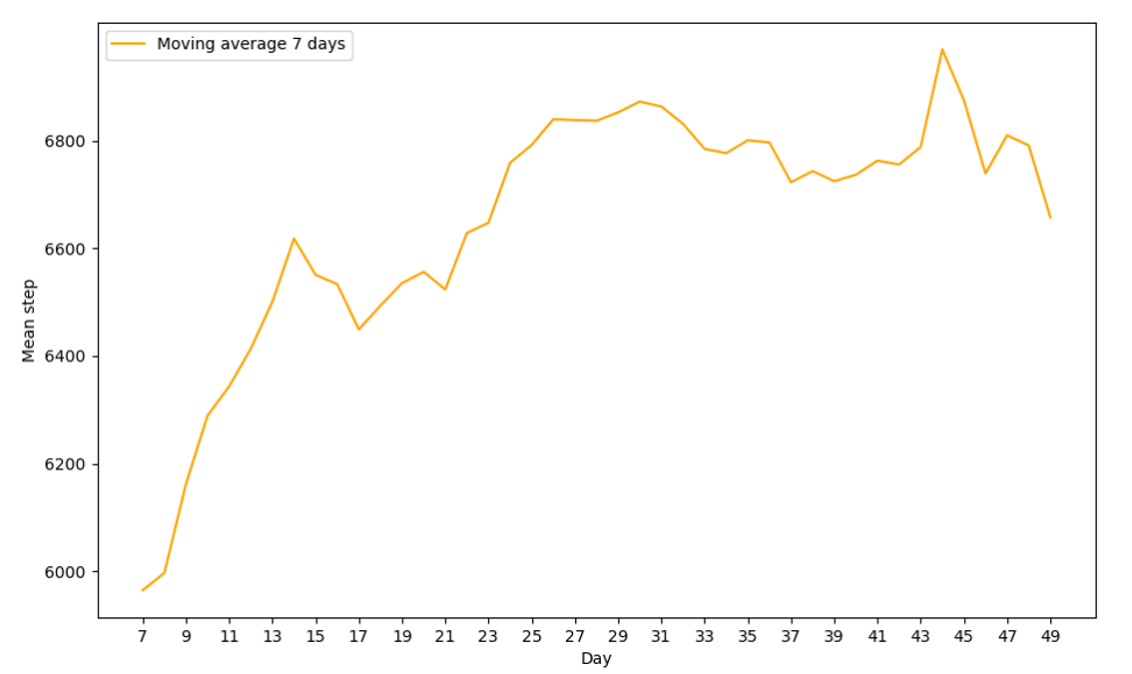
Change in mean daily step over time.

**Figure 4:**
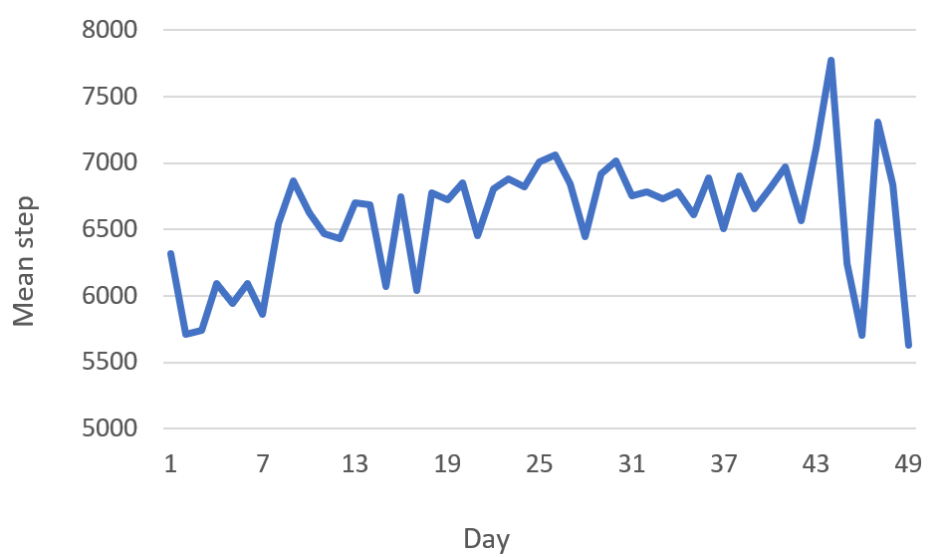
Change in mean daily step over time.

Figure 3 shows changes in the average number of Fitbit steps per day over the study period. The average steps increased throughout the study. Data for the first 7 days of Fitbit use, before receiving access to the chatbot, did not show an increase in mean step (Figure 4).

Table 3 shows improvement in the outcomes between follow-up and baseline. For both samples, BMI was improved but was not statistically significant at follow-up compared with baseline. On average, participants recorded significantly more steps at follow-up compared with baseline in the full sample (increase of 627, 95% CI 219-1035 steps/day) and in the subsample that excludes those with <4 weeks of exposure to the chatbot (increase of 564, 95% CI 120-1009 steps/day). Similarly, the total physical activity minutes at follow-up were 3.58 (95% CI 2.28-5.63) times higher in the full sample and 4.17 (95% CI 2.55-6.80) times higher in the subsample than at baseline, representing an increase 154.2 and 176.6 min/week, respectively. Participants were also more likely to meet the physical activity guideline at follow-up compared with baseline in the full sample (OR 6.37, 95% CI 3.31-12.27) and in the subsample (OR 6.41, 95% CI 3.14-13.09).

**Table 3:** Differences in the outcomes between follow-up and baseline.

				Baseline				Follow-up				Crude estimate (95% CI)		Adjusted estimate (95% CI)^a^
				Participants, n		Value		Participants, n		Value				
**Full sample (N=116**)														
	BMI (kg/m^2^), mean (SD)			116		32.5 (8.0)		116		32.4 (8.0)		−0.08 (−0.34 to 0.17)		−0.13 (−0.37 to 0.11)
	Step counts/day, mean (SD)			108		5933 (2391)		102		6570 (2326)		633^b^ (242 to 1024)		627^b^ (219 to 1035)
	Total physical activity (min/week), mean (SD)^c^			116		86.5 (137.5)		116		240.7 (233.6)		4.04^d^ (2.59 to 6.29)		3.58^d^ (2.28 to 5.63)
	**Meeting physical activity guidelines^e^, n (%)**													
		No	116		100 (86.2)		116		54 (46.6)		1.0		1.0	
		Yes	116		16 (13.8)		116		62 (53.5)		7.18^d^ (3.89 to 13.24)		6.37^d^ (3.31 to 12.27)	
**Subsample (n=99); excludes those with <4 week of chatbot use**														
	BMI (kg/m^2^), mean (SD)			99		32.0 (8.1)		99		31.9 (8.2)		−0.08 (−0.37 to 0.21)		−0.13 (−0.4 to 0.14)
	Step counts/day, mean (SD)			94		5958 (2444)		89		6530 (2297)		576^b^ (153 to 998)		564^f^ (120 to 1009)
	Total physical activity (min/week), mean (SD)^c^			99		88.9 (147.0)		99		265.5 (240.5)		4.69^d^ (2.92 to 7.55)		4.17^d^ (2.55 to 6.80)
	**Meeting physical activity guidelines^e^, n (%)**													
		No	99		84 (84.9)		99		43 (43.4)		1.0		1.0	
		Yes	99		15 (15.1)		99		56 (56.6)		7.29^d^ (3.77 to 14.12)		6.41^d^ (3.14 to 13.09)	

^a^Adjusted for age: gender, marital status, years of schooling, ethnicity, household income, living area, work status, and work duration.

^b^*P*<.01.

^c^Estimates were converted back to ratios.

^d^*P*<.001.

^e^Estimates are odds ratios.

^f^*P*<.05.

## Discussion

### Principal Findings

This study examined the feasibility, usability, and effectiveness of a physical activity chatbot with natural language processing capability and adaptive goal setting delivered via the Facebook Messenger app. Significant improvements in both the step count and self-reported physical activity were observed. These findings are consistent with those from another Australian study examining a combined diet and physical activity chatbot using natural language processing [20]. The effect on self-reported physical activity in this study (approximately 160 min/week) was similar to an increase of approximately 110 min/week of physical activity measured by accelerometers after a 12-week intervention in another study [20]. However, the effect measured by the Fitbits appeared to be smaller, as the average step count only increased by approximately 600 steps/day or 4200 steps/week, which roughly corresponds to an increase of 42 minutes of physical activity per week (assuming it takes approximately 10 minutes to take 1000 steps) [20]. The differences between self-reported and objectively measured physical activity are likely due to recall and social desirability bias [31]. It is also worth noting that an increase of 1000 steps/day can help reduce all-cause mortality risk between 6% and 36%, although an increase of approximately 600 steps may not have a similar effect [32]. In addition, the technical issues experienced in this study might have reduced the effectiveness of the intervention and explained the differences between the 2 studies. Moreover, it is possible that a longer study duration would have resulted in higher effectiveness, and that 6 weeks was not long enough to demonstrate the full potential of the chatbot. Those in the subsample that excluded those who had <4 weeks of exposure to the chatbot, had a higher increase in the total physical activity minutes compared with the full sample, which indicates that more exposure might have resulted in better outcomes. Finally, we also found a study that used a chatbot to promote stair climbing, and it also reported a significant increase in physical activity after 12 weeks of intervention, although it is not clear how physical activity was measured and what the effect size was [33].

The findings showed that the participants liked the chatbot with some even asking for continuing to use it after they completed the 6-week trial. Nevertheless, usability for both the chatbot and the Fitbit was rated as *OK* for majority of the participants. For most usability and acceptability indicators, less than half of the participants provided answers in favor of the chatbot. This level of usability (SUS scores of 61.6) is comparable with the 2 psychological therapy chatbots with SUS scores of 63.6 and 57.0 [34] but lower than that of asthma management (SUS score of about 83) [35] and depression prevention chatbots (SUS score of approximately 75.7) [36]. A study by Nadarzynski et al [37] also showed higher acceptability, with 67% of participants who would like to use a health chatbot. The lower acceptability in this study is likely due to technical issues that most participants experienced during the study. Some technical issues related to the use of the Fitbit, including malfunctioning Fitbits and broken bands and cables, were expected and dealt with by the research team. Most of the other technical issues (eg, the chatbot stopped sending daily notifications and difficulties connecting to the chatbot) were fixed by the management company. However, one issue beyond the control of the management company that resulted in the end of the study was that Facebook changed its policy to block the chatbot sending out messages to participants who did not respond to the chatbot within 24 hours. Facebook implemented this new policy to prevent chatbots from spamming its Messenger app users, however, inadvertently disabled the functionality of the chatbot, which sent messages wanted by our participants. Despite our efforts, it was not possible to contact Facebook to explain and reverse the situation. The Facebook policy change offers the most likely explanation for the discrepancy between high engagement and low usability scores; that is, most participants used the chatbot until the end of the implementation, but at the time of the Facebook policy change, they experienced serious technical issues undermining the usability of the chatbot.

Previous studies have also shown higher usability of Fitbit use [38-40] compared with this study. A possible explanation might be that because of budgetary reasons, the research team was forced to use Fitbit Flex 1, which is an old model in this study. Apart from being outdated in terms of user expectations (newer models with better functionality dominate the market), the long shelf life of the Fitbits meant that battery and connectivity problems were more prevalent than normal. We recommend the use of newer and higher-quality activity trackers to increase the feasibility of future chatbot-based physical activity interventions. Furthermore, we recommend that future chatbots be hosted on flexible messaging platforms that can be contacted for assistance in dealing with similar issues should they arise. However, the disadvantage of using such platforms is that people may be less familiar with the platform and more reluctant to use them, as few of their friends and family are likely to use those messaging services. In addition, rather than relying on an external technical company to develop and host the chatbot, it would be better if the research team is capable of doing this by itself, so that upgrading chatbot functionality and responding to potential technical problems is faster and more efficient.

The results also showed that BMI did not significantly improve at follow-up. This finding is not surprising, as our study did not target weight loss and therefore, no direct activity related to weight loss or weight maintenance was delivered. This is different from the other Australian chatbot-based physical activity interventions, which showed a significant decrease in weight at week 12 [20]. However, that study also included a large dietary component and allowed more time (12 weeks) for weight loss to occur [20]. As this study was not designed to evaluate the effect of each component (diet and physical activity) separately, it is impossible to determine whether the improvement in weight was due to increases in physical activity. Furthermore, current evidence regarding the effects of physical activity and exercise on weight loss is not strong [41].

### Strengths and Limitations

This study has several strengths: (1) both objective and subjective measures of physical activity were used to obtain accurate and complementary data on the effectiveness of the intervention [42] and (2) a high retention rate means that selection bias due to loss to follow-up was likely minimal. However, this study also has limitations. First, as the study was designed as a quasi-experiment without a control group, it was not possible to control for unknown confounders. It is also likely that the increase in steps occurred just by using the Fitbit [43]. However, it is worth noting that step counts in the baseline week before the participants started using the chatbot did not increase (Figure 4). Second, the sample (majority were women and White with high BMI) was not representative of the broader Australian population, so generalizability of the findings may be limited, although external validity was not the main focus of the intervention. Third, the technical issues caused by Facebook’s policy changes, which were beyond the research team’s control, were likely responsible for a reduction in chatbots’ usability, acceptability, and effectiveness. Finally, the short duration of the intervention (6 weeks) is a limitation, and the effects of chatbot interventions with a longer duration need to be examined.

### Conclusions

The machine learning–based physical activity chatbot was able to significantly increase participants’ physical activity and was moderately accepted by the participants. However, a Facebook policy change undermined the chatbot functionality and indicated the need to use independent platforms for chatbot deployment so that this type of intervention could be successfully delivered.

Future studies with stronger designs, such as randomized controlled trials, in which the effect of the activity trackers can be isolated, are needed to confirm these findings. Research is also required to determine whether chatbot-based interventions could be effective for broader populations. Furthermore, technology to develop and evaluate more comprehensive chatbot interventions already exists. In addition to natural language processing, Fitbit integration and adaptive goal setting, it is possible to use deep reinforcement learning with feedback loops and integrate more real-time data sources (eg, GPS and weather data) to enable chatbots to personally tailor and continuously adapt cues to action to ensure the timing, frequency, context, and content are optimally suited for each participant. It is important that such comprehensive physical activity chatbots should be developed and evaluated in future studies.

## Abbreviations

mHealth: mobile health
OR: odds ratio
SUS: System Usability Scale
